# Effect of the intrinsic and extrinsic factors on the growth and development of young foals under subtropical conditions of Pakistan

**DOI:** 10.1371/journal.pone.0310784

**Published:** 2025-01-30

**Authors:** Muhammad Athar Chatha, Nisar Ahmad, Muhammad Athar Abbas, Muhammad Saadullah, Jawaria Ali Khan

**Affiliations:** 1 Department of Livestock Management, University of Veterinary and Animal Sciences, Lahore, Pakistan; 2 Pakistan Agriculture Research Council, Islamabad, Pakistan; 3 Department of Clinical Medicine & Surgery, University of Veterinary and Animal Sciences, Lahore, Pakistan; Benha University Faculty of Veterinary Medicine, EGYPT

## Abstract

This study was designed to explore the impact of intrinsic (breed of foal, age of dam, and age of foal at weaning) and extrinsic (season of birth and housing type) factors on the growth and survival of foals in the subtropical conditions of Pakistan. For the growth study, retrospective data analysis of foals (n = 150) born from purebred brood mares of Thoroughbred, Arabs, and Percheron breeds (n_1_, n_2_, and n_3_ = 50 each) was made. Six hundred and twenty-four (n = 624) foals born between 2020 to 2022 were observed for the study of foal survival rate. The survival of these foals till the age of one year was considered. To study the growth and development of foals, height, bone, and girth measurements were taken at multiple developmental stages (3, 6, 9, 12, 15, and 18 months of age). Statistical analysis revealed that late-weaned foals demonstrated superior growth metrics compared to early-weaned foals (P = 0.001) and sheltered housing conditions markedly enhanced growth parameters across all breeds and measurement intervals (*P* = 0.002). However, no significant effect of season (*P > 0*.*05*) on the growth measurements across breeds was found. Arab and Thoroughbred breeds demonstrated significant early growth advantages in foals from middle-aged dams, with marked differences in height, bone width, and girth; however, by 15 months, these differences were not statistically significant (*P > 0*.*05*). In contrast, Percheron foals showed consistent growth regardless of the dam’s age, suggesting breed-specific developmental influences (*P* = 0.885). Regarding the effects of extrinsic and intrinsic factors on foal survival, environmental conditions, and maternal age significantly impacted survival rates. Extreme winter conditions were associated with a notably lower survival probability (*P* = 0.002), and middle-aged dams exhibited significantly enhanced survival odds (*P* = 0.03). However, the influences of housing conditions and weaning age on survival were not statistically significant (*P > 0*.*05*), indicating these factors do not substantially affect foal survival within the first year. These results underscore the critical roles of weaning age, housing conditions, and age of dams in influencing foal growth and survival, highlighting the importance of tailored management practices in optimizing outcomes for the growth and development of young equines under subtropics.

## Introduction

Pakistan’s equine culture is deeply rooted in history and horsemanship, where horses have been integral to transportation, agriculture, sports, and recreation [1, 2]. In the subtropical region of Pakistan, the equine population includes both indigenous and exotic breeds that hold distinct socio-cultural importance [3–5]. The Arabian, Thoroughbred, and Percheron horses are prominent among the exotic breeds in Pakistan. The Arabian horse, with its roots deeply embedded in the Middle East and a history dating back over 2,000 years in desert environments, is celebrated for its endurance, beauty, grace, and ability to prosper in harsh conditions [2, 6]. In contrast, the Thoroughbred, cultivated by the British aristocracy, has been selectively bred for exceptional racing performance over centuries [3, 7, 8]. The Percheron, originating from France [9], is predominantly used in Pakistan for ceremonial and religious events [2].

Various factors influence the growth and development of broodmares, including weaning age, housing conditions, and climate. The timing of weaning is particularly crucial, as it dramatically affects foal growth. Early weaning may slow growth initially, whereas later weaning promotes faster growth rates. This phase is essential due to changes in nutritional needs, making proper nutrition and close monitoring to prevent nutritional imbalances and support optimal growth [10, 11].

Housing and management practices also play a significant role in the development of young equines. Suitable housing that offers sufficient space, proper ventilation, and protection from environmental stressors is crucial for their health and well-being [12]. Additionally, the season of birth impacts foal development, with factors like forage quality, availability, and environmental conditions such as temperature and humidity influencing nutritional status, activity levels, and energy expenditure. These seasonal changes can also affect immune system development, potentially altering a foal’s disease resistance [13, 14].

Additionally, effective housing and management practices significantly influence the growth of young equines, with key considerations including proper space, ventilation, cleanliness, and disease control. Providing adequate space for movement and well-designed stalls promotes musculoskeletal development, while vigilant hygiene practices reduce the risk of infectious diseases [15]. Addressing environmental stressors by providing shade and insulation ensures optimal growth, complemented by timely vaccinations and deworming [16]. Regular exercise and socialization in a controlled environment contribute to physical and psychological well-being, emphasizing the holistic impact of thoughtful housing and management practices on young equines’ development [15].

Equine development can be measured using a variety of metrics that assess physical growth, health, and behavioral maturity. Key physical measurements include height at the withers, weight, and body condition scoring to assess the horse’s fat and muscle composition. Additionally, specific measurements like girth and cannon bone width provide critical data on the horse’s overall body condition and bone development [15, 17]. Balanced nutrition of foals is mandatory as under, and over-feeding can negatively affect the growth and development of young equines [18].

Despite a sizeable equine population in Pakistan’s livestock economy, quality equine availability remains challenging, leading to annual imports of sports horses. Limited research on young foals’ growth and development impedes the optimization of equine athletes in subtropical conditions. Comprehensive research is needed to understand intrinsic and extrinsic factors influencing foal growth, aiding in the production of more equine athletes annually. Hence, the present study has been designed to provide insights into how intrinsic factors like breed and age of the dam and extrinsic factors, such as season of birth and housing type, influence the growth and survival of foals of three different breeds (Arab, Thoroughbred and Percheron) in subtropical conditions of Pakistan.

## Materials and methods

### Location of study and ethical approval

The study was conducted across three divisions in Pakistan: Sargodha (32.1566° N, 72.8043° E), Faisalabad (31.4504° N, 73.1350° E), and Sahiwal (30.6490° N, 73.1350° E). At each division, a well-established breeding stud was selected, and the study included foals born from brood mares registered at these studs. All brood mares and their foals were housed in individual stalls or boxes. The study was approved by the Ethical Review Committee of the University of Veterinary and Animal Sciences, Lahore, vide letter number DR/71 dated 19 March 2023.

### Animals

In the first part of study about the effects of the type of weaning, season of birth, housing management, and age of brood mares on the growth and development of young equines, retrospective data analysis of foals (n = 150) born from purebred brood mares of Thoroughbred, Arabs, and Percheron breeds (n1, n2 and n3 = 50 each) was made. In second part of study retrospective analysis of six hundred and twenty-four (n = 624) foals (of all breeds) born between 2020 and 2022 was made to evaluate the foal survival rate till the age of one year.

In the first part of study, height, bone, and girth measurements were taken at multiple developmental stages (3, 6, 9, 12, 15, and 18 months) to assess physical growth. The second part of the study was aimed to identify trends and factors influencing early mortality across various breeds without differentiating by breed type.

### Climatic conditions and season of the birth of foal

In our study, the seasonal year in Pakistan was categorized into two distinct classifications to analyze their impact on foal growth and survival. The first classification split the year into two groups: 1) Extreme Weather Seasons, which include extreme summer (June and July) and extreme winter (December and January), characterized by harsher conditions that potentially challenge foal survival and growth; and 2) Moderate Weather Seasons, encompassing the remaining months (February to May, August to November), which offer more favourable conditions for the horses. For a better understanding of results, the extreme weather and moderate season have been described as "season-1" and "season-2", respectively. The second classification for the survival analysis specifically delineated three periods: Extreme Summer (June and July), Extreme Winter (December and January), and Moderate (February to May, August to November), to closely observe how the foals cope with varying degrees of environmental stressors across different times of the year.

### Housing and management of foals

The foals included in the study were housed in one of two systems: the open housing system, where they and their mothers were kept in expansive, fenced pastures allowing free movement and ample social interaction, or the sheltered housing system, which provided enclosed foaling boxes within barns or sheds for protection from environmental elements. The open system had temporary shade available for the young foals. In contrast, the sheltered system enabled close health and safety monitoring, offering a controlled environment with structured ventilation and bedding.

### Weaning of foals

Foals included in the study were divided into two groups based on the age of weaning. The early weaning group was weaned at three months of age, whereas the late weaning group was weaned at six months. From birth until three months, foals in both groups had free access to their dam’s milk or a milk replacer, ensuring optimal early nutrition. Creep feeding was initiated when foals from each group reached three weeks of age. After three months, foals in the early weaning group transitioned to a creep feed regimen, including small, frequent meals comprising 14–16% protein and 10–12% fat, while receiving conventional feed. The late-weaning group remained on the same creep feed formula, in addition to their regular feed, until they reached six months of age.

### Measurement of height, girth, and bone development of foals

Specific methodologies were employed to accurately measure young foals’ height, girth, and bone development. Height was measured using a horse measuring stick with a level positioned beside the foal on a flat surface. The stick was aligned with the highest point of the withers, and the measurement was taken where the horizontal bar touched this point. For girth measurements, a flexible measuring tape was wrapped snugly around the foal’s body at the heart girth, directly behind the forelimbs, to ensure accuracy without compressing the body. Bone development was assessed by measuring the width of the cannon bone, positioned between the knee and fetlock joint of the forelimbs, using a measuring tape. In addition, the circumference of this area was also measured. Measurements were taken at regular intervals and each measurement was repeated twice to ensure consistency and reliability.

### Foal survival

A retrospective analysis of the data of foals from three breeding studs for 2020–2022 was conducted. The study examined foal mortality during the first year of life without distinguishing between different breeds. The analysis focused on several independent variables, including the season of birth, housing type, weaning age, and dam’s age, to assess their impact on foal survival. This part of the study was meant to evaluate how these factors contribute to the likelihood of survival in the early developmental stages of foals under the subtropical conditions of Pakistan.

### Study variables

For both of the studies, the type of weaning (early and late), type of housing (foaling box or closed housing / open housing with temporary shade), the season of the birth of the foal (extreme and moderate), and age of dam (young, middle, or old age) were independent variables. In the first part of the study, the dependent variables were the height, bone size, and girth measurements of foals at 3, 6, 9, 12, 15, and 18 months of age. In the second part, the dependent variable was the survival rate of the foals during their first year of life.

### Statistical analysis

The normality of data was checked by the Shapiro-Wilk test. To study the effects of the types of weaning, housing, and season of the birth of a foal on the height, bone, and girth of foals at the age of 3, 6, 9, 12, 15, and 18 months, independent sample t-test was applied with 95% confidence interval. The effects of the age of brood mares on the height, bone, and girth of foals at the ages of 3, 6, 9, 12, 15, and 18 months were analyzed by One-Way ANOVA. The foals of each breed (Arab, Thoroughbred, and Percheron) were analyzed separately. To analyze the effects of independent variables on foal survival rate, the foals from all breeds were grouped, and the effects of independent variables on foal survival rate were analyzed by binary logistic regression.

## Results

### Impact of weaning age on growth of foals

**Fig 1:**
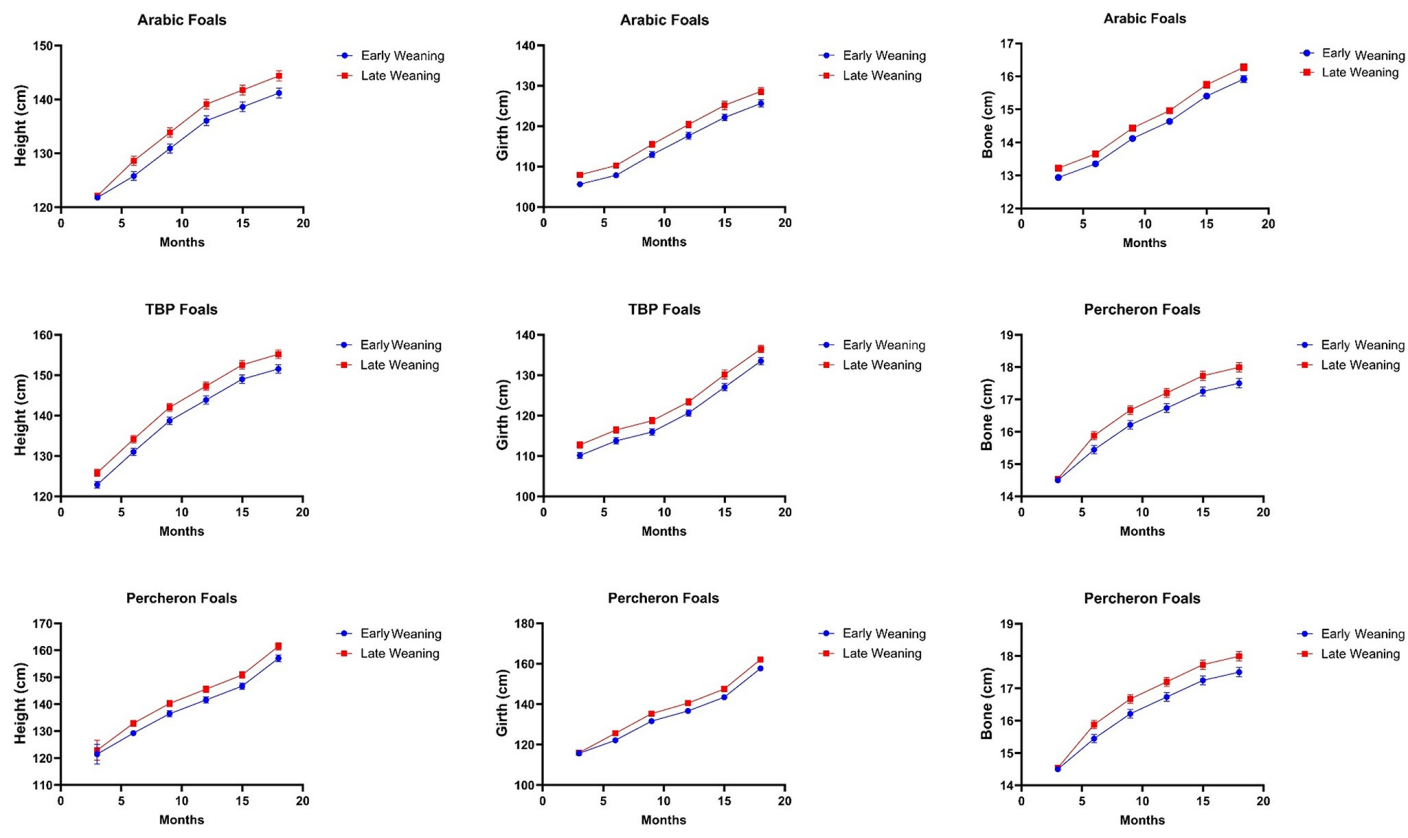
Effect of type of weaning on growth and development of Arab, Thoroughbred, and Percheron foals under subtropical conditions of Pakistan. S1 Table for detailed results.

The statistical analysis has revealed that late weaning significantly benefits foal growth compared to early weaning, with robust differences observed across all ages. In Arab foals, late-weaned individuals consistently displayed superior growth metrics. At three months, early-weaned Arab foals had a height of 121.81 ± 0.10 cm, compared to 122.15 ± 0.10 cm for late-weaned foals (*P = 0*.*001*). This trend of significant growth advantage in late-weaned foals continued through 18 months, where late-weaned Arab foals measured 144.37 ± 0.94 cm in height versus 141.20 ± 0.92 cm in early-weaned counterparts (*P = 0*.*001*). Similarly, in TBP and Percheron breeds, late weaning led to consistently higher growth measurements. At 18 months, late-weaned TBP foals had a height of 155.19 ± 1.08 cm, significantly taller than 151.57 ± 1.05 cm for early-weaned foals (*P = 0*.*001*). The results are summarized in Fig 1.

### Influence of housing conditions on growth of foals

**Fig 2:**
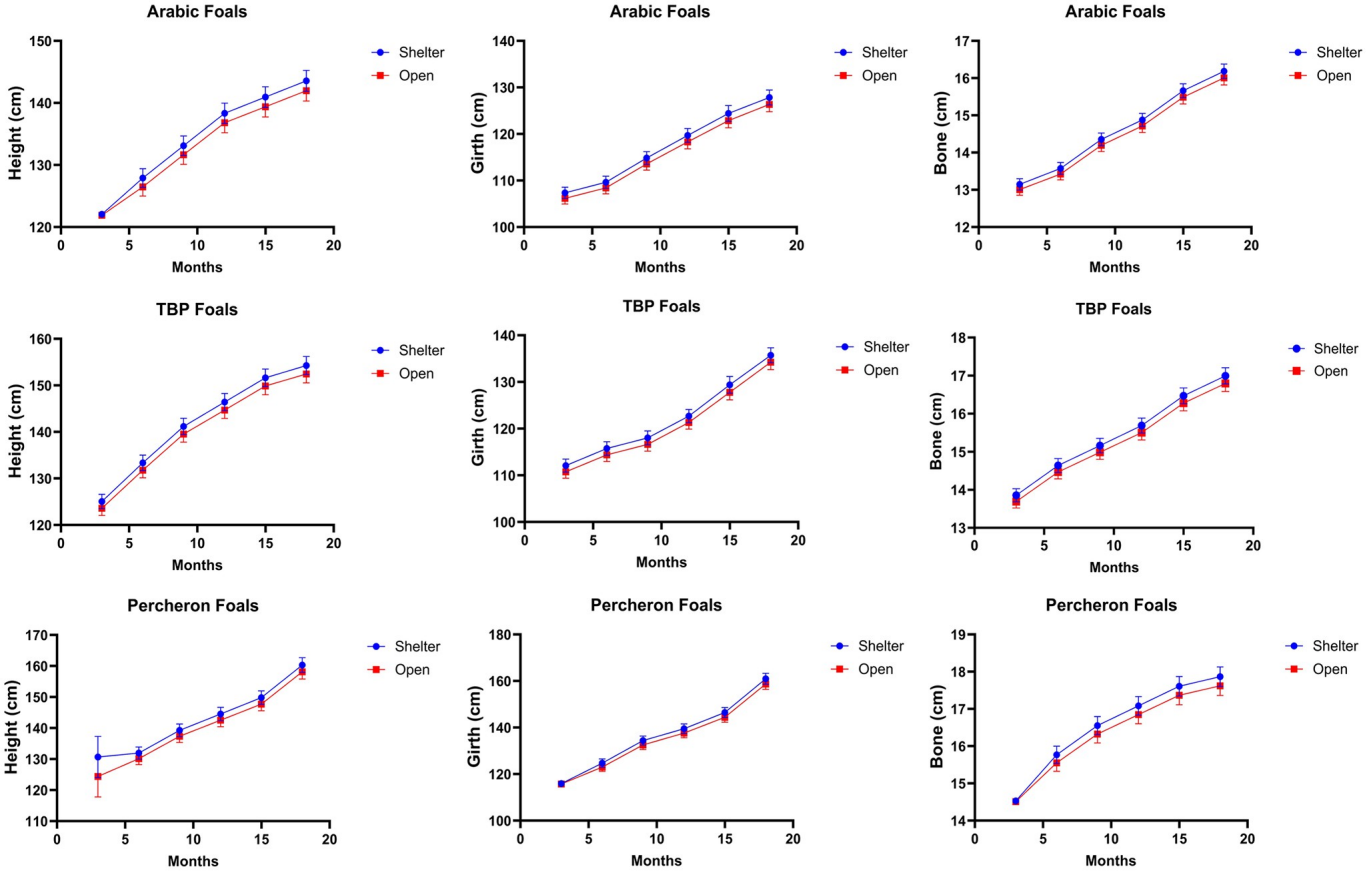
Effect of the type of housing on growth and development of Arab, Thoroughbred, and Percheron foals under subtropical conditions of Pakistan. S2 Table for detailed results.

The statistical analysis demonstrated that sheltered housing conditions significantly improved growth parameters in foals across all breeds and time intervals. Specifically, Arab foals housed in shelters at three months of age reached an average height of 122.06 ± 0.18 cm, compared to 121.89 ± 0.18 cm for those in open housing, showing a significant difference (P = 0.002). This growth advantage was sustained over time; by 18 months, the average height of shelter-housed Arab foals was 143.55 ± 1.70 cm, notably higher than the 141.96 ± 1.67 cm measured in their open-housed counterparts (P = 0.002). The trend was consistent in other breeds, too; for example, at 18 months, sheltered Percheron foals were significantly taller at 161.46 ± 1.32 cm compared to 157.04 ± 1.28 cm for those in open housing (*P = 0*.*002*). The results have been summarized in the Fig 2.

### Seasonal variation and growth impact

**Fig 3:**
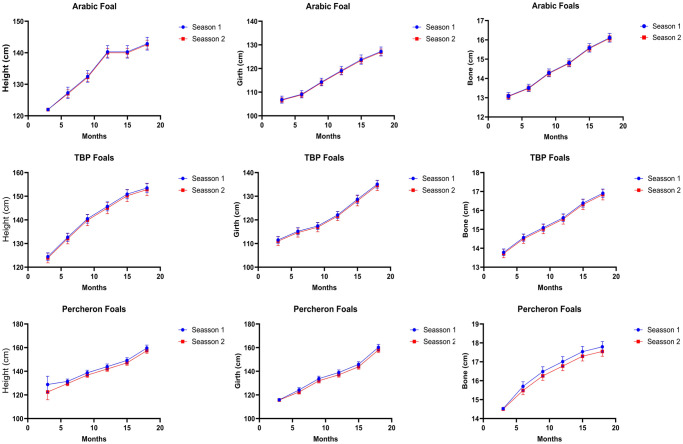
Effect of type of season on growth and development of Arab, Thoroughbred, and Percheron foals under subtropical conditions of Pakistan. S3 Table for detailed results.

Seasonal variations did not significantly affect the growth measurements across any breeds. For the Arab breed, the height of foals at three months was nearly identical across the two seasons: 121.99 ± 0.22 cm in Season 1 versus 121.96 ± 0.15 cm in Season 2 (*P = 0*.*608*). This lack of significant difference was consistent throughout all ages and breeds, indicating that seasonal factors might not play a critical role in influencing the physical growth parameters of foals. The results highlight that management practices such as optimal weaning age and housing conditions substantially impact foal growth, while seasonal variations have minimal effects on foal growth if housing, management and nutritional conditions are optimized. The results are summarized in Fig 3.

### Effect of the age of dam on growth and development of young foals

The statistical analysis of breed-wise data has revealed the impact of the dam’s age on the growth metrics of foals from three different horse breeds (Arab, Thoroughbred, and Percheron), providing a detailed view of developmental changes from 3 to 18 months.

In the Arab breed, foals from middle-aged dams showed superior growth measurements at three months: height was recorded at 122.08 ± 0.21 cm (P = 0.014), bone width at 13.16 ± 0.17 cm (P = 0.014), and girth at 107.42 ± 1.41 cm (P = 0.014). By six months, these advantages became even more pronounced, with heights reaching 128.35 ± 1.10 cm (P = 0.001), bone widths expanding to 13.62 ± 0.12 cm (P = 0.001), and girths increasing to 110.01 ± 0.94 cm (P = 0.001). However, by nine months, the growth metrics converged across all age groups, showing no significant differences (*P = 0*.*885*). Although the early advantages decrease as the foals age, modest benefits persisted at 15 months, particularly in height (141.44 ± 1.21 cm, *P = 0*.*001*) and girth (124.87 ± 1.31 cm, *P = 0*.*001*).

Similarly, Thoroughbred foals from middle-aged dams also exhibited an early growth advantage. At three months, significant differences in measurements were recorded for height (125.17 ± 1.79 cm, *P = 0*.*013*) and girth (112.17 ± 1.60 cm, *P = 0*.*013*), with this trend continuing at six months, particularly for height (133.84 ± 1.22 cm, *P = 0*.*001*) and girth (116.19 ± 1.06 cm, *P = 0*.*001*). However, by 15 months, the differences in growth metrics were no longer statistically significant, suggesting that other developmental factors become more influential over time.

**Fig 4:**
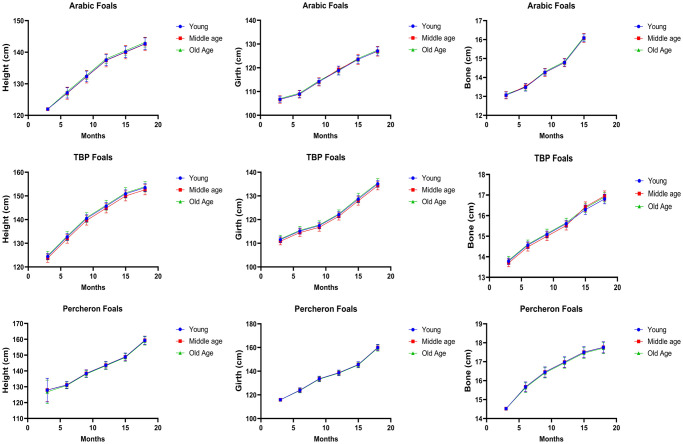
Effect of age of dam on growth and development of Arab, Thoroughbred, and Percheron foals under subtropical conditions of Pakistan. S4 Table of detailed results.

Conversely, the Percheron breed exhibited consistent growth patterns at all stages, showing no significant differences (P = 0.885). This suggests that, unlike in the Arab and Thoroughbred breeds, the age of the dam does not significantly influence early growth in Percherons foals. The results have been summarized in the Fig 4.

### Effect of extrinsic and intrinsic factors on foal survival during early age

The statistical analysis of data on foal survival revealed that environmental conditions and maternal age significantly impact foal survival rates. Foals born in extreme winter have a notably lower survival probability, with middle-aged dams also significantly enhancing survival odds. However, the effects of housing conditions and weaning age on survival were not statistically significant (*P<0*.*05*). Foals born in extreme summer conditions showed a lower survival rate of 90.4% (113/125) with an odds ratio (OR) of 2.007. Although this increase did not reach statistical significance, it indicates a potential trend that warrants further exploration (95% CI: 0.94 to 4.286, *P = 0*.*072*). In comparison, foals born during extreme winter conditions also exhibited a significantly lower survival rate of 87.5% (70/80), with an OR of 3.693, suggesting they are almost four times less likely to survive compared to those born in moderate conditions (95% CI: 1.587 to 8.596, *P = 0*.*002*).

Regarding housing conditions, foals in open housing demonstrated a survival rate of 91.2% (239/262) with an OR of 1.836, although this was not statistically significant (95% CI: 0.936 to 3.604, *P = 0*.*077*). Similarly, weaning age did not significantly influence survival, with early-weaned foals achieving a survival rate of 92.3% (241/261) and an OR of 1.087, showing that weaning timing does not substantially impact survival in the first year (95% CI: 0.571 to 2.066, *P = 0*.*8*).

However, survival rates varied significantly among foals from dams of different ages. Foals born to middle-aged dams had a survival rate of 89.4% (101 out of 113) with an odds ratio (OR) of 2.529, indicating a significantly lower likelihood of survival (95% CI: 1.096 to 5.836, p = 0.03). In contrast, foals from young and old dams experienced no significant survival disadvantages, maintaining high survival rates of 93.1% (229 out of 246) and 95.1% (251 out of 264), respectively.

These results underscore the influence of environmental conditions and dam’s age on foal survival. Despite the significantly higher survival odds during extreme winter and its lower percentages compared to summer, middle-aged dams’ beneficial effects highlight important considerations for mare breeders and veterinary practitioners aiming to enhance foal survival through optimized management practices. The results have been summarized in Table 1.

**Table 1:** Logistic regression model for the probability of survival in foals based on different seasonal and maternal factors.

Variables	Survival				P value	Odds ratio (OR)	95% C.I.for EXP(B)	
	Yes		No					
	N	N%	N	N%			Lower	Upper
**Dam age**								
Young	229	93.1	17	6.9	0.393	0.328	0.655	2.944
Middle age	101	89.4	12	10.6	**0.03**	0.928	1.096	5.836
Old age	251	95.1	13	4.9	0.09	**Reference**		
**Weather**								
Extreme Winter	70	87.5	10	12.5	**0.002**	1.307	1.587	8.596
Extreme summer season	113	90.4	12	9.6	0.072	0.697	0.94	4.286
Moderate	398	95	21	5	**0.007**	**Reference**		
**Weaning**								
Early weaning	241	92.3	20	7.7	0.8	0.083	0.571	2.066
Late weaning	340	93.7	23	6.3		**Reference**		
**Housing**								
Open	239	91.2	23	8.8	0.077	0.608	0.936	3.604
Shelter	342	94.5	20	5.5		**Reference**		

Note: The p value in bold shows significant results at α = 0.05.

## Discussion

The growth, body composition, and future athletic potential of young foals are shaped by a combination of intrinsic and extrinsic factors. Genetic predispositions, environmental influences, management strategies, and nutritional provisions all play crucial roles in their growth and developmental [15, 19]. The current study has evaluated evolving needs of the equine industry with the aim of optimizing foal development and survival rates. Through a retrospective analysis of both intrinsic and extrinsic factors that influence the growth and development of young foals, this research focuses on critical developmental stages from the first 24 hours up to 18 months. The findings suggest that late weaning contributes to superior growth outcomes compared to early weaning, as evidenced by significant differences in height, bone size, and girth across various breeds. A previous study has reported that foals weaned at six months should rely less on their dam’s milk and more on solid feed for their nutrients during weaning, indicating a critical period for nutritional transition [20, 21]. Another study noted that domesticated foals are typically weaned between four and seven months of age, with the weaning process being stressful for both mares and foals and requiring careful consideration [22–24].

While the current study suggests that late weaning leads to better growth outcomes, other studies have found that the optimal weaning age can vary depending on various factors, including the maturity of the foal, the health status and temperament of the mare and foal, and the design of the equine facility [25]. For instance, some horsemen wean at around three months, while others leave mare and foal together until the baby is four, five, or even six months old [10, 26]. This variability in weaning age is reflected in the natural weaning process, where spontaneous weaning of foals is thought to be mainly initiated by the dams when their foals are about 9 to 11 months old [10]. The better growth outcomes observed in late-weaned foals may be attributed to several factors. One possibility is that late weaning allows for a more gradual transition from milk to solid feed, reducing the stress associated with weaning and enabling foals to adapt more quickly to their new nutritional environment [27, 28].

Our study has revealed that foals in sheltered housing demonstrated superior growth metrics across all ages compared to those in open housing. For instance, by 18 months, Arab foals in sheltered environments were approximately 1.5 cm taller than those in open housing, consistent across various breeds. Conversely, a previous study has reported that unheated loose housing conditions do not increase respiratory diseases, provided they are well-managed [16]. This indicates that loose housing can be effectively utilized in harsh climates without adverse health impacts if proper management practices exist. Complementing these findings, another study by Autio & Heiskanen (2005) observed that foals spent significant time in varying environments within loose housing: 43.2% in the insulated sleeping hall, 51.4% in the open paddock, and 5.2% in a sheltered area indicating that foals are capable of adapting their behavior to different housing conditions [29]. Together, these studies illustrate that with adequate management, diverse housing systems can support both the health and growth of foals, highlighting young horses’ adaptability to various environmental conditions.

The better growth outcomes observed in our shelter-housed foals may be attributed to several factors. One possibility is that sheltered housing protects from environmental stressors, such as extreme temperatures and weather conditions, which can negatively impact foal growth [30]. Additionally, sheltered housing may allow for better management of nutrition and feeding practices, ensuring that foals receive the necessary nutrients for optimal growth. Furthermore, the social environment and stress levels associated with housing conditions may also play a role in foal growth, with sheltered housing potentially providing a more stable and less stressful environment [31].

Our study showed that seasonal changes have negligible effects on growth metrics across all breeds and ages, implying that seasonal variations do not significantly influence foal growth. However, these results contradict the existing literature, as studies have consistently demonstrated that seasonal changes significantly impact foal growth and development. For example, research has shown that foals born in winter are smaller than those born in summer, with apparent seasonal effects on foal size observed at birth and persisting up to 12 weeks of age [15]. This suggests that seasonal variations, particularly the reduced energy metabolism during winter, can affect fetal growth and development. Furthermore, studies have highlighted the importance of birth month and season on growth rate, with winter-born foals generally being smaller than those born later in the year [32, 33]. Another study showed that low late-summer precipitation during the females’ year of birth was associated with a pronounced decrease in foaling probability in response to harsh late-winter temperatures prior to the mating season [33]. The seasonal effects on foal growth may be attributed to various factors, including the reduced energy metabolism during winter, which can affect fetal growth and development. Additionally, the quality and availability of pasture, which can vary seasonally, can influence foal growth rates, with rapid daily weight gain observed in winter-born foals coinciding with a spring pasture flush. The relationships between mare body weight and condition, as well as foal growth, also suggest that seasonal effects on nutrition and energy availability can impact foal development [33, 34].

The age of the dam had a significant early impact on the growth of foals, especially in the Arab and Thoroughbred breeds. Middle-aged dams produced foals with better initial growth metrics, although these advantages decreased over time. This finding is supported by existing research about the growth rate of Thoroughbreds, and found that dams under seven years of age and older than 11 years had foals of lighter weight at birth compared to mares between 7 and 11 years of age, with these differences persisting at 510 days of age [35]. This suggests that middle-aged dams tend to produce foals with better initial growth metrics, which aligns with the statement. The study also found that foals born to dams in this age range had better growth rates, which could be attributed to the dams’ ability to provide better nutrition to the fetus during gestation.

Similarly, another study by Derisoud et al. showed that age essentially affects cyclicity, folliculogenesis, oocyte and embryo quality, oviductal masses, and uterine tract function. Maternal parity has a non-linear effect [36]. Primiparity significantly influences placental and foal development, with smaller foals in first gestation that remain smaller postnatally. After the first gestation, endometrial quality and uterine clearance capacities progressively decline with increasing parity, while placental and foal birthweight and milk production increase.

Environmental challenges, particularly extreme weather conditions, significantly impact foal mortality, with harsh winters being particularly detrimental. This correlation is supported by studies which emphasize the interactive importance of weather conditions experienced during early life and density and weather during current pregnancy on foaling probability, particularly in young females [33]. Similarly, another study from Canada has reported that the mortality rate is higher in foals born at the beginning of the year than in foals born later [37]. Furthermore, maternal factors, such as the age and health status of the dam, also play a crucial role in the survival of foals. Derisoud et al. (2021) found that the age and health of the broodmare significantly affect mortality rates, especially in the critical early months of a foal’s life [36].

## Conclusion

It can be divulged from the current study that the dam’s age, housing conditions, and weaning age critically influence foal growth and survival under subtropical conditions. Late weaning and sheltered environments significantly enhance growth metrics across breeds. Conversely, foals from middle-aged dams show reduced survival rates. Seasonal variations exert minimal influence, underscoring the importance of consistent management. These findings offer evidence-based recommendations for optimal foal development and management.

## Supporting information

S1 Table Impact of weaning type on the growth and development of Arab, Thoroughbred, and Percheron foals under subtropical conditions in Pakistan. The table presents mean values and standard deviations for height, bone, and girth measurements at 3, 6, 9, 12, 15, and 18 months of age. Statistically significant differences (P < 0.001) were observed between early and late weaning groups across all breeds and age intervals.
(DOCX)

S2 Table Effect of housing type on the growth and development of Arab, Thoroughbred, and Percheron foals under subtropical conditions in Pakistan. This table compares height, bone, and girth measurements of foals housed in sheltered versus open environments at various ages. Significant improvements (P = 0.002) in growth parameters were noted in foals housed in sheltered conditions across all age groups and breeds.
(DOCX)

S3 Table Influence of seasonal variation on the growth and development of Arab, Thoroughbred, and Percheron foals in subtropical regions of Pakistan. The table details height, bone, and girth measurements across two distinct seasons (Season-1 and Season-2). No significant differences were observed for Arab and Thoroughbred foals, suggesting minimal seasonal effects on growth when other conditions are held constant. Significant differences (P < 0.05) were observed in Percheron foals, highlighting the Impact of seasonal factors on their growth.
(DOCX)

S4 Table Influence of dam’s age on the growth and development of Arab, Thoroughbred, and Percheron foals in subtropical regions of Pakistan. The table compares growth metrics (height, bone, girth) in foals born to young, middle-aged, and older dams. Foals born to middle-aged dams showed significantly enhanced growth at 6, 12, 15, and 18 months (P < 0.05).
(DOCX)

S1 File This dataset contains detailed growth measurements for foals, including the dam’s age, foal breed, weaning type, housing conditions, and seasonality. It includes data on height, girth, and bone development at various ages under subtropical conditions. The dataset is structured to allow for comparison between early and late weaning, as well as between sheltered and open housing.
(XLSX)

S2 File This dataset provides survival rates and breed-specific mortality information for foals under subtropical conditions. It includes data on total young stock, early-age mortality by breed, and survival trends, emphasizing the influence of housing and environmental factors on foal survival.
(XLSX)
