# Supplementary material for: Effect of the intrinsic and extrinsic factors on the growth and development of young foals under subtropical conditions of Pakistan
Source: PLoS One. 2025 Jan 30;20(1):e0310784. doi: 10.1371/journal.pone.0310784 (PMC11781635; doi:10.1371/journal.pone.0310784)
Supplement: S1 Table — The table presents mean values and standard deviations for height, bone, and girth measurements at 3, 6, 9, 12, 15, and 18 months of age. Statistically significant differences (P < 0.001) were observed between early and late weaning groups across all breeds and age intervals. (DOCX) [file pone.0310784.s001.docx]

| **S1 Result Table: Effect of the Type of Weaning on the Growth and Development of Arab, Thoroughbred and Percheron Foals under subtropical conditions of Pakistan** | | | | | | | | | |
| --- | --- | --- | --- | --- | --- | --- | --- | --- | --- |
| **breed** | **foal age** |  | **weaning** | **N** | **Mean** | **Std. Deviation** | **t** | **df** | **Sig. (2-tailed)** |
| Arab | 3 Months | Height | Early | 25 | 121.8123 | 0.099607 | -12.01 | 48 | 0.000 |
|  |  |  | Late Weaning | 25 | 122.1512 | 0.099884 |  |  |  |
|  |  | Bone | Early | 25 | 12.9368 | 0.083902 | -12.01 | 48 | 0.000 |
|  |  |  | Late Weaning | 25 | 13.2218 | 0.083902 |  |  |  |
|  |  | Girth | Early | 25 | 105.616 | 0.684462 | -12.01 | 48 | 0.000 |
|  |  |  | Late Weaning | 25 | 107.941 | 0.684462 |  |  |  |
|  | 6 Months | Height | Early | 25 | 125.7967 | 0.822599 | -12.008 | 48 | 0.000 |
|  |  |  | Late Weaning | 25 | 128.6222 | 0.841075 |  |  |  |
|  |  | Bone | Early | 25 | 13.35187 | 0.087309 | -12.008 | 48 | 0.000 |
|  |  |  | Late Weaning | 25 | 13.65177 | 0.08927 |  |  |  |
|  |  | Girth | Early | 25 | 107.8257 | 0.705084 | -12.008 | 48 | 0.000 |
|  |  |  | Late Weaning | 25 | 110.2476 | 0.720921 |  |  |  |
|  | 9 Months | Height | Early | 25 | 130.9312 | 0.856174 | -12.008 | 48 | 0.000 |
|  |  |  | Late Weaning | 25 | 133.8721 | 0.875404 |  |  |  |
|  |  | Bone | Early | 25 | 14.12003 | 0.092333 | -12.008 | 48 | 0.000 |
|  |  |  | Late Weaning | 25 | 14.43718 | 0.094406 |  |  |  |
|  |  | Girth | Early | 25 | 112.9603 | 0.73866 | -12.008 | 48 | 0.000 |
|  |  |  | Late Weaning | 25 | 115.4975 | 0.755251 |  |  |  |
|  | 12 Months | Height | Early | 25 | 136.0658 | 0.889749 | -12.008 | 48 | 0.000 |
|  |  |  | Late Weaning | 25 | 139.1219 | 0.909734 |  |  |  |
|  |  | Bone | Early | 25 | 14.63551 | 0.095703 | -12.008 | 48 | 0.000 |
|  |  |  | Late Weaning | 25 | 14.96424 | 0.097853 |  |  |  |
|  |  | Girth | Early | 25 | 117.6074 | 0.817091 | -12.008 | 48 | 0.000 |
|  |  |  | Late Weaning | 25 | 120.416 | 0.836603 |  |  |  |
|  | 15 Months | Height | Early | 25 | 138.6331 | 0.906537 | -12.008 | 48 | 0.000 |
|  |  |  | Late Weaning | 25 | 141.7469 | 0.926899 |  |  |  |
|  |  | Bone | Early | 25 | 15.40367 | 0.100726 | -12.008 | 48 | 0.000 |
|  |  |  | Late Weaning | 25 | 15.74965 | 0.102989 |  |  |  |
|  |  | Girth | Early | 25 | 122.1588 | 0.848712 | -10.952 | 48 | 0.000 |
|  |  |  | Late Weaning | 25 | 125.156 | 1.07337 |  |  |  |
|  | 18 Months | Height | Early | 25 | 141.2003 | 0.923325 | -12.008 | 48 | 0.000 |
|  |  |  | Late Weaning | 25 | 144.3718 | 0.944064 |  |  |  |
|  |  | Bone | Early | 25 | 15.91915 | 0.104097 | -12.008 | 48 | 0.000 |
|  |  |  | Late Weaning | 25 | 16.27671 | 0.106435 |  |  |  |
|  |  | Girth | Early | 25 | 125.6178 | 0.872744 | -12.008 | 48 | 0.000 |
|  |  |  | Late Weaning | 25 | 128.6177 | 0.893586 |  |  |  |
| TBP | 3 Months | Height | Early | 25 | 122.887 | 0.853771 | -12.008 | 48 | 0.000 |
|  |  |  | Late Weaning | 25 | 125.8216 | 0.87416 |  |  |  |
|  |  | Bone | Early | 25 | 13.61366 | 0.094582 | -12.008 | 48 | 0.000 |
|  |  |  | Late Weaning | 25 | 13.93876 | 0.096841 |  |  |  |
|  |  | Girth | Early | 25 | 110.123 | 0.765091 | -12.008 | 48 | 0.000 |
|  |  |  | Late Weaning | 25 | 112.7528 | 0.783362 |  |  |  |
|  | 6 Months | Height | Early | 25 | 131.0188 | 0.910268 | -12.008 | 48 | 0.000 |
|  |  |  | Late Weaning | 25 | 134.1476 | 0.932005 |  |  |  |
|  |  | Bone | Early | 25 | 14.38233 | 0.099923 | -12.008 | 48 | 0.000 |
|  |  |  | Late Weaning | 25 | 14.72579 | 0.102309 |  |  |  |
|  |  | Girth | Early | 25 | 113.7337 | 0.790178 | -12.008 | 48 | 0.000 |
|  |  |  | Late Weaning | 25 | 116.4497 | 0.809047 |  |  |  |
|  | 9 Months | Height | Early | 25 | 138.7258 | 0.963813 | -12.008 | 48 | 0.000 |
|  |  |  | Late Weaning | 25 | 142.0387 | 0.986829 |  |  |  |
|  |  | Bone | Early | 25 | 14.89816 | 0.103507 | -12.008 | 48 | 0.000 |
|  |  |  | Late Weaning | 25 | 15.25393 | 0.105978 |  |  |  |
|  |  | Girth | Early | 25 | 115.9689 | 0.805707 | -12.008 | 48 | 0.000 |
|  |  |  | Late Weaning | 25 | 118.7384 | 0.824948 |  |  |  |
|  | 12 Months | Height | Early | 25 | 143.8638 | 0.99951 | -12.008 | 48 | 0.000 |
|  |  |  | Late Weaning | 25 | 147.2993 | 1.023378 |  |  |  |
|  |  | Bone | Early | 25 | 15.41398 | 0.10709 | -12.008 | 48 | 0.000 |
|  |  |  | Late Weaning | 25 | 15.78207 | 0.109648 |  |  |  |
|  |  | Girth | Early | 25 | 120.6621 | 0.789023 | -12.008 | 48 | 0.000 |
|  |  |  | Late Weaning | 25 | 123.3723 | 0.806745 |  |  |  |
|  | 15 Months | Height | Early | 25 | 149.0018 | 1.035206 | -12.008 | 48 | 0.000 |
|  |  |  | Late Weaning | 25 | 152.56 | 1.059928 |  |  |  |
|  |  | Bone | Early | 25 | 16.18265 | 0.112431 | -12.008 | 48 | 0.000 |
|  |  |  | Late Weaning | 25 | 16.56911 | 0.115116 |  |  |  |
|  |  | Girth | Early | 25 | 127.0452 | 0.88266 | -10.952 | 48 | 0.000 |
|  |  |  | Late Weaning | 25 | 130.1623 | 1.116305 |  |  |  |
|  | 18 Months | Height | Early | 25 | 151.5708 | 1.053055 | -12.008 | 48 | 0.000 |
|  |  |  | Late Weaning | 25 | 155.1904 | 1.078202 |  |  |  |
|  |  | Bone | Early | 25 | 16.69848 | 0.116015 | -12.008 | 48 | 0.000 |
|  |  |  | Late Weaning | 25 | 17.09725 | 0.118785 |  |  |  |
|  |  | Girth | Early | 25 | 133.4985 | 0.872962 | -12.008 | 48 | 0.000 |
|  |  |  | Late Weaning | 25 | 136.497 | 0.892569 |  |  |  |
| Percheron | 3 Months | Height | Early | 25 | 121.4161 | 3.688276 | -11.964 | 48 | 0.000 |
|  |  |  | Late Weaning | 25 | 133.8967 | 3.688276 |  |  |  |
|  |  | Bone | Early | 25 | 14.49932 | 0.011856 | -12.01 | 48 | 0.000 |
|  |  |  | Late Weaning | 25 | 14.53965 | 0.011889 |  |  |  |
|  |  | Girth | Early | 25 | 115.6541 | 0.094572 | -12.01 | 48 | 0.000 |
|  |  |  | Late Weaning | 25 | 115.9758 | 0.094835 |  |  |  |
|  | 6 Months | Height | Early | 25 | 129.2551 | 1.056397 | -12.008 | 48 | 0.000 |
|  |  |  | Late Weaning | 25 | 132.8938 | 1.086136 |  |  |  |
|  |  | Bone | Early | 25 | 15.44494 | 0.126231 | -12.008 | 48 | 0.000 |
|  |  |  | Late Weaning | 25 | 15.87974 | 0.129785 |  |  |  |
|  |  | Girth | Early | 25 | 122.1711 | 0.9985 | -12.008 | 48 | 0.000 |
|  |  |  | Late Weaning | 25 | 125.6104 | 1.026609 |  |  |  |
|  | 9 Months | Height | Early | 25 | 136.4303 | 1.11504 | -12.008 | 48 | 0.000 |
|  |  |  | Late Weaning | 25 | 140.271 | 1.14643 |  |  |  |
|  |  | Bone | Early | 25 | 16.21516 | 0.132526 | -12.008 | 48 | 0.000 |
|  |  |  | Late Weaning | 25 | 16.67164 | 0.136257 |  |  |  |
|  |  | Girth | Early | 25 | 131.6266 | 1.075779 | -12.008 | 48 | 0.000 |
|  |  |  | Late Weaning | 25 | 135.332 | 1.106064 |  |  |  |
|  | 12 Months | Height | Early | 25 | 141.5786 | 1.157117 | -12.008 | 48 | 0.000 |
|  |  |  | Late Weaning | 25 | 145.5643 | 1.189691 |  |  |  |
|  |  | Bone | Early | 25 | 16.73202 | 0.13675 | -12.008 | 48 | 0.000 |
|  |  |  | Late Weaning | 25 | 17.20305 | 0.1406 |  |  |  |
|  |  | Girth | Early | 25 | 136.6634 | 1.116945 | -12.008 | 48 | 0.000 |
|  |  |  | Late Weaning | 25 | 140.5107 | 1.148389 |  |  |  |
|  | 15 Months | Height | Early | 25 | 146.7269 | 1.199194 | -12.008 | 48 | 0.000 |
|  |  |  | Late Weaning | 25 | 150.8575 | 1.232953 |  |  |  |
|  |  | Bone | Early | 25 | 17.24888 | 0.140975 | -12.008 | 48 | 0.000 |
|  |  |  | Late Weaning | 25 | 17.73446 | 0.144943 |  |  |  |
|  |  | Girth | Early | 25 | 143.4535 | 1.17244 | -12.008 | 48 | 0.000 |
|  |  |  | Late Weaning | 25 | 147.4919 | 1.205446 |  |  |  |
|  | 18 Months | Height | Early | 25 | 157.0438 | 1.283514 | -12.008 | 48 | 0.000 |
|  |  |  | Late Weaning | 25 | 161.4648 | 1.319646 |  |  |  |
|  |  | Bone | Early | 25 | 17.50224 | 0.143045 | -12.008 | 48 | 0.000 |
|  |  |  | Late Weaning | 25 | 17.99495 | 0.147072 |  |  |  |
|  |  | Girth | Early | 25 | 157.6519 | 1.288483 | -12.008 | 48 | 0.000 |
|  |  |  | Late Weaning | 25 | 162.09 | 1.324756 |  |  |  |
