# Supplementary material for: Effect of the intrinsic and extrinsic factors on the growth and development of young foals under subtropical conditions of Pakistan
Source: PLoS One. 2025 Jan 30;20(1):e0310784. doi: 10.1371/journal.pone.0310784 (PMC11781635; doi:10.1371/journal.pone.0310784)
Supplement: S2 Table — This table compares height, bone, and girth measurements of foals housed in sheltered versus open environments at various ages. Significant improvements (P = 0.002) in growth parameters were noted in foals housed in sheltered conditions across all age groups and breeds. (DOCX) [file pone.0310784.s002.docx]

| **S2 Result Table: Effect of the Type of Housing on the on the Growth and Development of Arab, Thoroughbred and Percheron Foals under subtropical conditions of Pakistan** | | | | | | | | | |
| --- | --- | --- | --- | --- | --- | --- | --- | --- | --- |
| **breed** | **foal age** |  | **housing** | **N** | **Mean** | **Std. Deviation** | **t** | **df** | **Sig. (2-tailed)** |
| Arab | 3 Months | Height | Shelter | 26 | 122.0631 | 0.180459 | 3.326 | 48 | 0.002 |
|  |  |  | Open | 24 | 121.8937 | 0.179401 |  |  |  |
|  |  | Bone | Shelter | 26 | 13.1477 | 0.151693 | 3.326 | 48 | 0.002 |
|  |  |  | Open | 24 | 13.0052 | 0.151014 |  |  |  |
|  |  | Girth | Shelter | 26 | 107.3365 | 1.237494 | 3.326 | 48 | 0.002 |
|  |  |  | Open | 24 | 106.174 | 1.231954 |  |  |  |
|  | 6 Months | Height | Shelter | 26 | 127.8875 | 1.511919 | 3.325 | 48 | 0.002 |
|  |  |  | Open | 24 | 126.4749 | 1.488524 |  |  |  |
|  |  | Bone | Shelter | 26 | 13.57379 | 0.160473 | 3.325 | 48 | 0.002 |
|  |  |  | Open | 24 | 13.42386 | 0.15799 |  |  |  |
|  |  | Girth | Shelter | 26 | 109.6178 | 1.295931 | 3.325 | 48 | 0.002 |
|  |  |  | Open | 24 | 108.407 | 1.275877 |  |  |  |
|  | 9 Months | Height | Shelter | 26 | 133.1074 | 1.57363 | 3.325 | 48 | 0.002 |
|  |  |  | Open | 24 | 131.6371 | 1.54928 |  |  |  |
|  |  | Bone | Shelter | 26 | 14.35472 | 0.169705 | 3.325 | 48 | 0.002 |
|  |  |  | Open | 24 | 14.19616 | 0.167079 |  |  |  |
|  |  | Girth | Shelter | 26 | 114.8377 | 1.357641 | 3.325 | 48 | 0.002 |
|  |  |  | Open | 24 | 113.5693 | 1.336633 |  |  |  |
|  | 12 Months | Height | Shelter | 26 | 138.3273 | 1.635341 | 3.325 | 48 | 0.002 |
|  |  |  | Open | 24 | 136.7994 | 1.610036 |  |  |  |
|  |  | Bone | Shelter | 26 | 14.87876 | 0.175901 | 3.325 | 48 | 0.002 |
|  |  |  | Open | 24 | 14.71442 | 0.173179 |  |  |  |
|  |  | Girth | Shelter | 26 | 119.6857 | 1.503342 | 3.325 | 48 | 0.002 |
|  |  |  | Open | 24 | 118.2816 | 1.479052 |  |  |  |
|  | 15 Months | Height | Shelter | 26 | 140.9372 | 1.666196 | 3.325 | 48 | 0.002 |
|  |  |  | Open | 24 | 139.3805 | 1.640414 |  |  |  |
|  |  | Bone | Shelter | 26 | 15.65969 | 0.185133 | 3.325 | 48 | 0.002 |
|  |  |  | Open | 24 | 15.48672 | 0.182268 |  |  |  |
|  |  | Girth | Shelter | 26 | 124.3944 | 1.714958 | 3.325 | 48 | 0.002 |
|  |  |  | Open | 24 | 122.859 | 1.536291 |  |  |  |
|  | 18 Months | Height | Shelter | 26 | 143.5471 | 1.697052 | 3.325 | 48 | 0.002 |
|  |  |  | Open | 24 | 141.9616 | 1.670792 |  |  |  |
|  |  | Bone | Shelter | 26 | 16.18373 | 0.191328 | 3.325 | 48 | 0.002 |
|  |  |  | Open | 24 | 16.00498 | 0.188368 |  |  |  |
|  |  | Girth | Shelter | 26 | 127.8376 | 1.605737 | 3.325 | 48 | 0.002 |
|  |  |  | Open | 24 | 126.3379 | 1.579793 |  |  |  |
| TBP | 3 Months | Height | Shelter | 26 | 125.0586 | 1.570829 | 3.325 | 48 | 0.002 |
|  |  |  | Open | 24 | 123.5914 | 1.545449 |  |  |  |
|  |  | Bone | Shelter | 26 | 13.85422 | 0.174019 | 3.325 | 48 | 0.002 |
|  |  |  | Open | 24 | 13.69169 | 0.171208 |  |  |  |
|  |  | Girth | Shelter | 26 | 112.0689 | 1.40767 | 3.325 | 48 | 0.002 |
|  |  |  | Open | 24 | 110.7542 | 1.384926 |  |  |  |
|  | 6 Months | Height | Shelter | 26 | 133.334 | 1.674776 | 3.325 | 48 | 0.002 |
|  |  |  | Open | 24 | 131.7698 | 1.647716 |  |  |  |
|  |  | Bone | Shelter | 26 | 14.63648 | 0.183845 | 3.325 | 48 | 0.002 |
|  |  |  | Open | 24 | 14.46477 | 0.180875 |  |  |  |
|  |  | Girth | Shelter | 26 | 115.7435 | 1.453825 | 3.325 | 48 | 0.002 |
|  |  |  | Open | 24 | 114.3856 | 1.430336 |  |  |  |
|  | 9 Months | Height | Shelter | 26 | 141.1772 | 1.773292 | 3.325 | 48 | 0.002 |
|  |  |  | Open | 24 | 139.521 | 1.744641 |  |  |  |
|  |  | Bone | Shelter | 26 | 15.16142 | 0.190439 | 3.325 | 48 | 0.002 |
|  |  |  | Open | 24 | 14.98355 | 0.187362 |  |  |  |
|  |  | Girth | Shelter | 26 | 118.0182 | 1.482397 | 3.325 | 48 | 0.002 |
|  |  |  | Open | 24 | 116.6337 | 1.458446 |  |  |  |
|  | 12 Months | Height | Shelter | 26 | 146.406 | 1.838969 | 3.325 | 48 | 0.002 |
|  |  |  | Open | 24 | 144.6884 | 1.809257 |  |  |  |
|  |  | Bone | Shelter | 26 | 15.68636 | 0.197032 | 3.325 | 48 | 0.002 |
|  |  |  | Open | 24 | 15.50233 | 0.193849 |  |  |  |
|  |  | Girth | Shelter | 26 | 122.6676 | 1.450208 | 3.325 | 48 | 0.002 |
|  |  |  | Open | 24 | 121.3126 | 1.427768 |  |  |  |
|  | 15 Months | Height | Shelter | 26 | 151.6348 | 1.904647 | 3.325 | 48 | 0.002 |
|  |  |  | Open | 24 | 149.8559 | 1.873873 |  |  |  |
|  |  | Bone | Shelter | 26 | 16.46862 | 0.206858 | 3.325 | 48 | 0.002 |
|  |  |  | Open | 24 | 16.27541 | 0.203516 |  |  |  |
|  |  | Girth | Shelter | 26 | 129.3702 | 1.783556 | 3.325 | 48 | 0.002 |
|  |  |  | Open | 24 | 127.7734 | 1.597743 |  |  |  |
|  | 18 Months | Height | Shelter | 26 | 154.2492 | 1.937485 | 3.325 | 48 | 0.002 |
|  |  |  | Open | 24 | 152.4396 | 1.906182 |  |  |  |
|  |  | Bone | Shelter | 26 | 16.99355 | 0.213452 | 3.325 | 48 | 0.002 |
|  |  |  | Open | 24 | 16.79419 | 0.210003 |  |  |  |
|  |  | Girth | Shelter | 26 | 135.7173 | 1.604485 | 3.325 | 48 | 0.002 |
|  |  |  | Open | 24 | 134.2182 | 1.579658 |  |  |  |
| Percheron | 3 Months | Height | Shelter | 26 | 130.6564 | 6.639512 | 3.325 | 48 | 0.002 |
|  |  |  | Open | 24 | 124.4064 | 6.628083 |  |  |  |
|  |  | Bone | Shelter | 26 | 14.52917 | 0.02148 | 3.325 | 48 | 0.002 |
|  |  |  | Open | 24 | 14.509 | 0.021354 |  |  |  |
|  |  | Girth | Shelter | 26 | 115.8922 | 0.171336 | 3.325 | 48 | 0.002 |
|  |  |  | Open | 24 | 115.7313 | 0.170332 |  |  |  |
|  | 6 Months | Height | Shelter | 26 | 131.9476 | 1.949647 | 3.325 | 48 | 0.002 |
|  |  |  | Open | 24 | 130.1285 | 1.914157 |  |  |  |
|  |  | Bone | Shelter | 26 | 15.76668 | 0.232967 | 3.325 | 48 | 0.002 |
|  |  |  | Open | 24 | 15.54931 | 0.228726 |  |  |  |
|  |  | Girth | Shelter | 26 | 124.7161 | 1.842794 | 3.325 | 48 | 0.002 |
|  |  |  | Open | 24 | 122.9967 | 1.809249 |  |  |  |
|  | 9 Months | Height | Shelter | 26 | 139.2723 | 2.057876 | 3.325 | 48 | 0.002 |
|  |  |  | Open | 24 | 137.3522 | 2.020415 |  |  |  |
|  |  | Bone | Shelter | 26 | 16.55294 | 0.244585 | 3.325 | 48 | 0.002 |
|  |  |  | Open | 24 | 16.32473 | 0.240133 |  |  |  |
|  |  | Girth | Shelter | 26 | 134.3685 | 1.985417 | 3.325 | 48 | 0.002 |
|  |  |  | Open | 24 | 132.516 | 1.949276 |  |  |  |
|  | 12 Months | Height | Shelter | 26 | 144.5279 | 2.135531 | 3.325 | 48 | 0.002 |
|  |  |  | Open | 24 | 142.5353 | 2.096658 |  |  |  |
|  |  | Bone | Shelter | 26 | 17.08057 | 0.252381 | 3.325 | 48 | 0.002 |
|  |  |  | Open | 24 | 16.84508 | 0.247787 |  |  |  |
|  |  | Girth | Shelter | 26 | 139.5103 | 2.061391 | 3.325 | 48 | 0.002 |
|  |  |  | Open | 24 | 137.5869 | 2.023867 |  |  |  |
|  | 15 Months | Height | Shelter | 26 | 149.7834 | 2.213187 | 3.325 | 48 | 0.002 |
|  |  |  | Open | 24 | 147.7184 | 2.1729 |  |  |  |
|  |  | Bone | Shelter | 26 | 17.60819 | 0.260177 | 3.325 | 48 | 0.002 |
|  |  |  | Open | 24 | 17.36544 | 0.255441 |  |  |  |
|  |  | Girth | Shelter | 26 | 146.4418 | 2.163811 | 3.325 | 48 | 0.002 |
|  |  |  | Open | 24 | 144.4229 | 2.124423 |  |  |  |
|  | 18 Months | Height | Shelter | 26 | 160.3152 | 2.368804 | 3.325 | 48 | 0.002 |
|  |  |  | Open | 24 | 158.105 | 2.325684 |  |  |  |
|  |  | Bone | Shelter | 26 | 17.86683 | 0.263999 | 3.325 | 48 | 0.002 |
|  |  |  | Open | 24 | 17.62051 | 0.259193 |  |  |  |
|  |  | Girth | Shelter | 26 | 160.936 | 2.377976 | 3.325 | 48 | 0.002 |
|  |  |  | Open | 24 | 158.7172 | 2.334689 |  |  |  |
