# Supplementary material for: Effect of the intrinsic and extrinsic factors on the growth and development of young foals under subtropical conditions of Pakistan
Source: PLoS One. 2025 Jan 30;20(1):e0310784. doi: 10.1371/journal.pone.0310784 (PMC11781635; doi:10.1371/journal.pone.0310784)
Supplement: S3 Table — The table details height, bone, and girth measurements across two distinct seasons (Season-1 and Season-2). No significant differences were observed for Arab and Thoroughbred foals, suggesting minimal seasonal effects on growth when other conditions are held constant. Significant differences (P < 0.05) were observed in Percheron foals, highlighting the Impact of seasonal factors on their growth. (DOCX) [file pone.0310784.s003.docx]

| **S3 Result Table: Effect of Season on the Growth and Development of Arab, Thoroughbred and Percheron Foals under subtropical conditions of Pakistan** | | | | | | | | | |
| --- | --- | --- | --- | --- | --- | --- | --- | --- | --- |
| **breed** | **foal age** |  | **Season** | **N** | **Mean** | **Std. Deviation** | **t** | **df** | **Sig. (2-tailed)** |
| Arab | 3 Months | Height | Season-1 | 35 | 121.9913 | 0.215921 | 0.516 | 48 | 0.608 |
|  |  |  | Season-2 | 15 | 121.9595 | 0.150435 |  |  |  |
|  |  | Bone | Season-1 | 35 | 13.08728 | 0.18164 | 0.515 | 48 | 0.609 |
|  |  |  | Season-2 | 15 | 13.06068 | 0.126496 |  |  |  |
|  |  | Girth | Season-1 | 35 | 106.8436 | 1.481799 | 0.515 | 48 | 0.609 |
|  |  |  | Season-2 | 15 | 106.6266 | 1.031939 |  |  |  |
|  | 6 Months | Height | Season-1 | 35 | 127.2905 | 1.799539 | 0.527 | 48 | 0.6 |
|  |  |  | Season-2 | 15 | 127.0203 | 1.257581 |  |  |  |
|  |  | Bone | Season-1 | 35 | 13.51042 | 0.191 | 0.527 | 48 | 0.6 |
|  |  |  | Season-2 | 15 | 13.48174 | 0.133478 |  |  |  |
|  |  | Girth | Season-1 | 35 | 109.1061 | 1.542462 | 0.527 | 48 | 0.6 |
|  |  |  | Season-2 | 15 | 108.8745 | 1.077927 |  |  |  |
|  | 9 Months | Height | Season-1 | 35 | 132.486 | 1.872989 | 0.527 | 48 | 0.6 |
|  |  |  | Season-2 | 15 | 132.2048 | 1.308911 |  |  |  |
|  |  | Bone | Season-1 | 35 | 14.28771 | 0.201989 | 0.527 | 48 | 0.6 |
|  |  |  | Season-2 | 15 | 14.25738 | 0.141157 |  |  |  |
|  |  | Girth | Season-1 | 35 | 114.3017 | 1.615912 | 0.527 | 48 | 0.6 |
|  |  |  | Season-2 | 15 | 114.059 | 1.129257 |  |  |  |
|  | 12 Months | Height | Season-1 | 35 | 137.6815 | 1.94644 | 0.527 | 48 | 0.6 |
|  |  |  | Season-2 | 15 | 137.3893 | 1.360241 |  |  |  |
|  |  | Bone | Season-1 | 35 | 14.80931 | 0.209363 | 0.527 | 48 | 0.6 |
|  |  |  | Season-2 | 15 | 14.77787 | 0.14631 |  |  |  |
|  |  | Girth | Season-1 | 35 | 119.0924 | 1.788661 | 0.528 | 48 | 0.6 |
|  |  |  | Season-2 | 15 | 118.8234 | 1.25025 |  |  |  |
|  | 15 Months | Height | Season-1 | 35 | 140.2793 | 1.983165 | 0.527 | 48 | 0.6 |
|  |  |  | Season-2 | 15 | 139.9815 | 1.385906 |  |  |  |
|  |  | Bone | Season-1 | 35 | 15.58659 | 0.220352 | 0.527 | 48 | 0.6 |
|  |  |  | Season-2 | 15 | 15.5535 | 0.15399 |  |  |  |
|  |  | Girth | Season-1 | 35 | 123.7584 | 1.973587 | 0.605 | 48 | 0.548 |
|  |  |  | Season-2 | 15 | 123.4219 | 1.298634 |  |  |  |
|  | 18 Months | Height | Season-1 | 35 | 142.8771 | 2.01989 | 0.527 | 48 | 0.6 |
|  |  |  | Season-2 | 15 | 142.5738 | 1.411571 |  |  |  |
|  |  | Bone | Season-1 | 35 | 16.10819 | 0.227726 | 0.527 | 48 | 0.6 |
|  |  |  | Season-2 | 15 | 16.074 | 0.159143 |  |  |  |
|  |  | Girth | Season-1 | 35 | 127.204 | 1.910489 | 0.528 | 48 | 0.6 |
|  |  |  | Season-2 | 15 | 126.9167 | 1.335406 |  |  |  |
| TBP | 3 Months | Height | Season-1 | 41 | 124.4578 | 1.631544 | 0.911 | 48 | 0.367 |
|  |  |  | Season-2 | 9 | 123.8831 | 2.078745 |  |  |  |
|  |  | Bone | Season-1 | 41 | 13.78767 | 0.180746 | 0.911 | 48 | 0.367 |
|  |  |  | Season-2 | 9 | 13.72401 | 0.230287 |  |  |  |
|  |  | Girth | Season-1 | 41 | 111.5306 | 1.462078 | 0.911 | 48 | 0.367 |
|  |  |  | Season-2 | 9 | 111.0156 | 1.862829 |  |  |  |
|  | 6 Months | Height | Season-1 | 41 | 132.6935 | 1.739508 | 0.911 | 48 | 0.367 |
|  |  |  | Season-2 | 9 | 132.0808 | 2.216302 |  |  |  |
|  |  | Bone | Season-1 | 41 | 14.56617 | 0.190951 | 0.911 | 48 | 0.367 |
|  |  |  | Season-2 | 9 | 14.49891 | 0.24329 |  |  |  |
|  |  | Girth | Season-1 | 41 | 115.1875 | 1.510018 | 0.911 | 48 | 0.367 |
|  |  |  | Season-2 | 9 | 114.6556 | 1.923908 |  |  |  |
|  | 9 Months | Height | Season-1 | 41 | 140.499 | 1.841832 | 0.911 | 48 | 0.367 |
|  |  |  | Season-2 | 9 | 139.8503 | 2.346672 |  |  |  |
|  |  | Bone | Season-1 | 41 | 15.08858 | 0.1978 | 0.911 | 48 | 0.367 |
|  |  |  | Season-2 | 9 | 15.01892 | 0.252016 |  |  |  |
|  |  | Girth | Season-1 | 41 | 117.4513 | 1.539694 | 0.911 | 48 | 0.367 |
|  |  |  | Season-2 | 9 | 116.909 | 1.961719 |  |  |  |
|  | 12 Months | Height | Season-1 | 41 | 145.7027 | 1.910048 | 0.911 | 48 | 0.367 |
|  |  |  | Season-2 | 9 | 145.0299 | 2.433586 |  |  |  |
|  |  | Bone | Season-1 | 41 | 15.611 | 0.204648 | 0.911 | 48 | 0.367 |
|  |  |  | Season-2 | 9 | 15.53892 | 0.260741 |  |  |  |
|  |  | Girth | Season-1 | 41 | 122.1128 | 1.506704 | 0.911 | 48 | 0.367 |
|  |  |  | Season-2 | 9 | 121.5818 | 1.919877 |  |  |  |
|  | 15 Months | Height | Season-1 | 41 | 150.9063 | 1.978264 | 0.911 | 48 | 0.367 |
|  |  |  | Season-2 | 9 | 150.2096 | 2.5205 |  |  |  |
|  |  | Bone | Season-1 | 41 | 16.3895 | 0.214854 | 0.911 | 48 | 0.367 |
|  |  |  | Season-2 | 9 | 16.31383 | 0.273744 |  |  |  |
|  |  | Girth | Season-1 | 41 | 128.7198 | 1.803079 | 0.939 | 48 | 0.352 |
|  |  |  | Season-2 | 9 | 128.075 | 2.149084 |  |  |  |
|  | 18 Months | Height | Season-1 | 41 | 153.5082 | 2.012372 | 0.911 | 48 | 0.367 |
|  |  |  | Season-2 | 9 | 152.7994 | 2.563957 |  |  |  |
|  |  | Bone | Season-1 | 41 | 16.91192 | 0.221702 | 0.911 | 48 | 0.367 |
|  |  |  | Season-2 | 9 | 16.83383 | 0.28247 |  |  |  |
|  |  | Girth | Season-1 | 41 | 135.1035 | 1.666991 | 0.911 | 48 | 0.367 |
|  |  |  | Season-2 | 9 | 134.516 | 2.12412 |  |  |  |
| Percheron | 3 Months | Height | Season-1 | 41 | 128.8023 | 6.992121 | 2.498 | 48 | 0.016 |
|  |  |  | Season-2 | 9 | 122.4363 | 6.570141 |  |  |  |
|  |  | Bone | Season-1 | 41 | 14.52313 | 0.02268 | 2.452 | 48 | 0.018 |
|  |  |  | Season-2 | 9 | 14.50291 | 0.020935 |  |  |  |
|  |  | Girth | Season-1 | 41 | 115.844 | 0.180908 | 2.452 | 48 | 0.018 |
|  |  |  | Season-2 | 9 | 115.6828 | 0.166991 |  |  |  |
|  | 6 Months | Height | Season-1 | 41 | 131.402 | 2.049608 | 2.445 | 48 | 0.018 |
|  |  |  | Season-2 | 9 | 129.5823 | 1.875012 |  |  |  |
|  |  | Bone | Season-1 | 41 | 15.70148 | 0.244912 | 2.445 | 48 | 0.018 |
|  |  |  | Season-2 | 9 | 15.48404 | 0.224049 |  |  |  |
|  |  | Girth | Season-1 | 41 | 124.2003 | 1.937277 | 2.445 | 48 | 0.018 |
|  |  |  | Season-2 | 9 | 122.4804 | 1.772249 |  |  |  |
|  | 9 Months | Height | Season-1 | 41 | 138.6964 | 2.163386 | 2.445 | 48 | 0.018 |
|  |  |  | Season-2 | 9 | 136.7757 | 1.979097 |  |  |  |
|  |  | Bone | Season-1 | 41 | 16.48449 | 0.257125 | 2.445 | 48 | 0.018 |
|  |  |  | Season-2 | 9 | 16.25621 | 0.235222 |  |  |  |
|  |  | Girth | Season-1 | 41 | 133.8129 | 2.087213 | 2.445 | 48 | 0.018 |
|  |  |  | Season-2 | 9 | 131.9598 | 1.909413 |  |  |  |
|  | 12 Months | Height | Season-1 | 41 | 143.9302 | 2.245024 | 2.445 | 48 | 0.018 |
|  |  |  | Season-2 | 9 | 141.937 | 2.05378 |  |  |  |
|  |  | Bone | Season-1 | 41 | 17.00993 | 0.265321 | 2.445 | 48 | 0.018 |
|  |  |  | Season-2 | 9 | 16.77438 | 0.24272 |  |  |  |
|  |  | Girth | Season-1 | 41 | 138.9334 | 2.167082 | 2.445 | 48 | 0.018 |
|  |  |  | Season-2 | 9 | 137.0094 | 1.982478 |  |  |  |
|  | 15 Months | Height | Season-1 | 41 | 149.164 | 2.326661 | 2.445 | 48 | 0.018 |
|  |  |  | Season-2 | 9 | 147.0984 | 2.128463 |  |  |  |
|  |  | Bone | Season-1 | 41 | 17.53538 | 0.273517 | 2.445 | 48 | 0.018 |
|  |  |  | Season-2 | 9 | 17.29255 | 0.250217 |  |  |  |
|  |  | Girth | Season-1 | 41 | 145.8362 | 2.274754 | 2.445 | 48 | 0.018 |
|  |  |  | Season-2 | 9 | 143.8167 | 2.080978 |  |  |  |
|  | 18 Months | Height | Season-1 | 41 | 159.6523 | 2.490257 | 2.445 | 48 | 0.018 |
|  |  |  | Season-2 | 9 | 157.4414 | 2.278123 |  |  |  |
|  |  | Bone | Season-1 | 41 | 17.79295 | 0.277534 | 2.445 | 48 | 0.018 |
|  |  |  | Season-2 | 9 | 17.54655 | 0.253893 |  |  |  |
|  |  | Girth | Season-1 | 41 | 160.2705 | 2.499899 | 2.445 | 48 | 0.018 |
|  |  |  | Season-2 | 9 | 158.051 | 2.286944 |  |  |  |
