# Supplementary material for: Effect of the intrinsic and extrinsic factors on the growth and development of young foals under subtropical conditions of Pakistan
Source: PLoS One. 2025 Jan 30;20(1):e0310784. doi: 10.1371/journal.pone.0310784 (PMC11781635; doi:10.1371/journal.pone.0310784)
Supplement: S4 Table — The table compares growth metrics (height, bone, girth) in foals born to young, middle-aged, and older dams. Foals born to middle-aged dams showed significantly enhanced growth at 6, 12, 15, and 18 months (P < 0.05). (DOCX) [file pone.0310784.s004.docx]

**S4 Result Table:**  **Effect of age of Dam on Growth and Development of Arab, Thoroughbred and Percheron Foals under Subtropical Conditions of Pakistan.**

|  | | | | | |  |
| --- | --- | --- | --- | --- | --- | --- |
| **Breed** | **foal age** | **Parameter** | **Age of Dam** | **N** | **Mean** | **P-Value** |
| Arab | 3 Months | Height | Young | 14 | 121.91+0.18 | .013 |
|  |  |  | Middle Age | 21 | 122.08+0.21 | .013 |
|  |  |  | Old | 15 | 121.92+0.15 | .941 |
|  |  | Bone | Young | 14 | 13.02+0.15 | .013 |
|  |  |  | Middle Age | 21 | 13.16+0.17 | .013 |
|  |  |  | Old | 15 | 13.02+0.13 | .940 |
|  |  | Girth | Young | 14 | 106.29+1.23 | .013 |
|  |  |  | Middle Age | 21 | 107.42+1.41 | .013 |
|  |  |  | Old | 15 | 106.33+1.06 | .940 |
| Arab | 6 Months | Height | Young | 11 | 126.24+1.51 | .000 |
|  |  |  | Middle Age | 26 | 128.35+1.10 | .000 |
|  |  |  | Old | 13 | 125.75+0.84 | .305 |
|  |  | Bone | Young | 11 | 13.40+0.16 | .000 |
|  |  |  | Middle Age | 26 | 13.62+0.12 | .000 |
|  |  |  | Old | 13 | 13.35+0.09 | .305 |
|  |  | Girth | Young | 11 | 108.21+1.30 | .000 |
|  |  |  | Middle Age | 26 | 110.01+0.94 | .000 |
|  |  |  | Old | 13 | 107.79+0.72 | .305 |
| Arab | 9 Months | Height | Young | 13 | 132.44+1.77 | .937 |
|  |  |  | Middle Age | 24 | 132.49+.74 | .937 |
|  |  |  | Old | 13 | 132.20+1.73 | .723 |
|  |  | Bone | Young | 13 | 14.28+0.19 | .937 |
|  |  |  | Middle Age | 24 | 14.29+0.19 | .937 |
|  |  |  | Old | 13 | 14.26+0.19 | .723 |
|  |  | Girth | Young | 13 | 114.26+1.53 | .937 |
|  |  |  | Middle Age | 24 | 114.31+1.50 | .937 |
|  |  |  | Old | 13 | 114.05+1.50 | .723 |
| Arab | 12 Months | Height | Young | 14 | 136.96+1.61 | .012 |
|  |  |  | Middle Age | 21 | 138.44+1.86 | .012 |
|  |  |  | Old | 15 | 137.00+1.38 | .943 |
|  |  | Bone | Young | 14 | 14.73+0.17 | .012 |
|  |  |  | Middle Age | 21 | 14.89+0.20 | .012 |
|  |  |  | Old | 15 | 14.74+0.15 | .943 |
|  |  | Girth | Young | 14 | 118.42+1.48 | .012 |
|  |  |  | Middle Age | 21 | 119.79+1.71 | .012 |
|  |  |  | Old | 15 | 118.47+1.27 | .943 |
| Arab | 15 Months | Height | Young | 11 | 139.12+1.67 | .000 |
|  |  |  | Middle Age | 26 | 141.44+1.21 | .000 |
|  |  |  | Old | 13 | 138.59+0.92 | .305 |
|  |  | Bone | Young | 11 | 15.46+0.19 | .000 |
|  |  |  | Middle Age | 26 | 15.72+0.13 | .000 |
|  |  |  | Old | 13 | 15.40+0.10 | .305 |
|  |  | Girth | Young | 11 | 122.62+1.56 | .000 |
|  |  |  | Middle Age | 26 | 124.87+1.31 | .000 |
|  |  |  | Old | 13 | 122.11+0.86 | .341 |
| Arab | 18 Months | Height | Young | 11 | 141.70+1.70 | .000 |
|  |  |  | Middle Age | 26 | 144.06+1.23 | .000 |
|  |  |  | Old | 13 | 141.15+0.94 | .305 |
|  |  | Bone | Young | 11 | 15.98+0.19 | .000 |
|  |  |  | Middle Age | 26 | 16.24+0.14 | .000 |
|  |  |  | Old | 13 | 15.91+0.11 | .305 |
|  |  | Girth | Young | 11 | 126.09+1.61 | .000 |
|  |  |  | Middle Age | 26 | 128.33+1.17 | .000 |
|  |  |  | Old | 13 | 125.57+0.89 | .304 |
| TBP | 3 Months | Height | Young | 14 | 123.74+1.54 | .012 |
|  |  |  | Middle Age | 21 | 125.17+1.79 | .012 |
|  |  |  | Old | 15 | 123.78+1.32 | .943 |
|  |  | Bone | Young | 14 | 13.71+0.17 | .012 |
|  |  |  | Middle Age | 21 | 13.87+0.20 | .012 |
|  |  |  | Old | 15 | 13.71+0.15 | .943 |
|  |  | Girth | Young | 14 | 110.89+1.38 | .012 |
|  |  |  | Middle Age | 21 | 112.17+1.60 | .012 |
|  |  |  | Old | 15 | 110.93+1.19 | .943 |
| TBP | 6 Months | Height | Young | 11 | 131.51+1.68 | .000 |
|  |  |  | Middle Age | 26 | 133.84+1.22 | .000 |
|  |  |  | Old | 13 | 130.97+0.93 | .304 |
|  |  | Bone | Young | 11 | 14.44+0.18 | .000 |
|  |  |  | Middle Age | 26 | 14.69+0.13 | .000 |
|  |  |  | Old | 13 | 14.38+0.10 | .304 |
|  |  | Girth | Young | 11 | 114.16+1.46 | .000 |
|  |  |  | Middle Age | 26 | 116.19+1.06 | .000 |
|  |  |  | Old | 13 | 113.69+0.80 | .304 |
| TBP | 9 Months | Height | Young | 13 | 140.43+1.99 | .937 |
|  |  |  | Middle Age | 24 | 140.48+1.96 | .937 |
|  |  |  | Old | 13 | 140.15+1.95 | .723 |
|  |  | Bone | Young | 13 | 15.08+0.21 | .937 |
|  |  |  | Middle Age | 24 | 15.09+0.21 | .937 |
|  |  |  | Old | 13 | 15.05+0.21 | .723 |
|  |  | Girth | Young | 13 | 117.39+1.67 | .937 |
|  |  |  | Middle Age | 24 | 117.44+1.64 | .937 |
|  |  |  | Old | 13 | 117.16+1.63 | .723 |
| TBP | 12 Months | Height | Young | 14 | 144.86+1.81 | .012 |
|  |  |  | Middle Age | 21 | 146.54+2.09 | .012 |
|  |  |  | Old | 15 | 144.91+1.55 | .943 |
|  |  | Bone | Young | 14 | 15.52+0.19 | .012 |
|  |  |  | Middle Age | 21 | 15.70+0.22 | .012 |
|  |  |  | Old | 15 | 15.53+0.17 | .943 |
|  |  | Girth | Young | 14 | 121.45+1.42 | .012 |
|  |  |  | Middle Age | 21 | 122.77+1.65 | .012 |
|  |  |  | Old | 15 | 121.49+1.22 | .943 |
| TBP | 15 Months | Height | Young | 12 | 150.89+1.63 | .252 |
|  |  |  | Middle Age | 16 | 149.99+2.10 | .252 |
|  |  |  | Old | 22 | 151.29+2.18 | .587 |
|  |  | Bone | Young | 12 | 16.39+0.18 | .252 |
|  |  |  | Middle Age | 16 | 16.29+0.23 | .252 |
|  |  |  | Old | 22 | 16.43+0.24 | .587 |
|  |  | Girth | Young | 8 | 128.85+1.42 | .350 |
|  |  |  | Middle Age | 13 | 128.05+1.85 | .350 |
|  |  |  | Old | 22 | 129.09+2.04 | .751 |
| TBP | 18 Months | Height | Young | 13 | 153.43+2.18 | .937 |
|  |  |  | Middle Age | 24 | 153.49+2.14 | .937 |
|  |  |  | Old | 13 | 153.13+2.13 | .723 |
|  |  | Bone | Young | 13 | 16.90+0.24 | .937 |
|  |  |  | Middle Age | 24 | 16.91+0.24 | .937 |
|  |  |  | Old | 13 | 16.87+0.24 | .723 |
|  |  | Girth | Young | 13 | 135.04+1.81 | .937 |
|  |  |  | Middle Age | 24 | 135.09+1.77 | .937 |
|  |  |  | Old | 13 | 134.79+1.77 | .723 |
| Percheron | 3 Months | Height | Young | 13 | 127.89+7.51 | .967 |
|  |  |  | Middle Age | 24 | 127.99+7.38 | .967 |
|  |  |  | Old | 13 | 126.81+7.40 | .713 |
|  |  | Bone | Young | 13 | 14.52+0.02 | .937 |
|  |  |  | Middle Age | 24 | 14.52+0.02 | .937 |
|  |  |  | Old | 13 | 14.52+0.02 | .723 |
|  |  | Girth | Young | 13 | 115.82+0.19 | .937 |
|  |  |  | Middle Age | 24 | 115.82+0.19 | .937 |
|  |  |  | Old | 13 | 115.79+0.19 | .723 |
| Percheron | 6 Months | Height | Young | 13 | 131.12+2.19 | .937 |
|  |  |  | Middle Age | 24 | 131.18+2.15 | .937 |
|  |  |  | Old | 13 | 130.82+2.14 | .723 |
|  |  | Bone | Young | 13 | 15.67+0.26 | .937 |
|  |  |  | Middle Age | 24 | 15.68+0.26 | .937 |
|  |  |  | Old | 13 | 15.63+0.26 | .723 |
|  |  | Girth | Young | 13 | 123.94+2.07 | .937 |
|  |  |  | Middle Age | 24 | 123.99+2.04 | .937 |
|  |  |  | Old | 13 | 123.65+2.03 | .723 |
| Percheron | 9 Months | Height | Young | 13 | 138.40+2.31 | .937 |
|  |  |  | Middle Age | 24 | 138.47+2.27 | .937 |
|  |  |  | Old | 13 | 138.08+2.26 | .723 |
|  |  | Bone | Young | 13 | 16.45+0.27 | .937 |
|  |  |  | Middle Age | 24 | 16.46+0.27 | .937 |
|  |  |  | Old | 13 | 16.41+0.27 | .723 |
|  |  | Girth | Young | 13 | 133.53+2.23 | .937 |
|  |  |  | Middle Age | 24 | 133.59+2.19 | .937 |
|  |  |  | Old | 13 | 133.22+2.18 | .723 |
| Percheron | 12 Months | Heigh | Young | 13 | 143.63+2.40 | .937 |
|  |  |  | Middle Age | 24 | 143.69+2.36 | .937 |
|  |  |  | Old | 13 | 143.30+2.35 | .723 |
|  |  | Bone | Young | 13 | 16.97+0.28 | .937 |
|  |  |  | Middle Age | 24 | 16.98+0.28 | .937 |
|  |  |  | Old | 13 | 16.93+0.28 | .723 |
|  |  | Girth | Young | 13 | 138.64+2.32 | .937 |
|  |  |  | Middle Age | 24 | 138.70+2.28 | .937 |
|  |  |  | Old | 13 | 138.32+2.27 | .723 |
| Percheron | 15 Months | Height | Young | 13 | 148.85+2.49 | .937 |
|  |  |  | Middle Age | 24 | 148.92+2.44 | .937 |
|  |  |  | Old | 13 | 148.51+2.43 | .723 |
|  |  | Bone | Young | 13 | 17.50+0.29 | .937 |
|  |  |  | Middle Age | 24 | 17.51+0.29 | .937 |
|  |  |  | Old | 13 | 17.46+0.29 | .723 |
|  |  | Girth | Young | 13 | 145.53+2.43 | .937 |
|  |  |  | Middle Age | 24 | 145.59+2.39 | .937 |
|  |  |  | Old | 13 | 145.19+2.38 | .723 |
| Percheron | 18 Months | Height | Young | 13 | 159.32+2.66 | .937 |
|  |  |  | Middle Age | 24 | 159.39+2.62 | .937 |
|  |  |  | Old | 13 | 158.95+2.60 | .723 |
|  |  | Bone | Young | 13 | 17.76+0.30 | .937 |
|  |  |  | Middle Age | 24 | 17.76+0.29 | .937 |
|  |  |  | Old | 13 | 17.71+0.29 | .723 |
|  |  | Girth | Young | 13 | 159.93+2.67 | .937 |
|  |  |  | Middle Age | 24 | 160.00+2.63 | .937 |
|  |  |  | Old | 13 | 159.56+2.61 | .723 |
